# Experimental Study on Basic Mechanical Properties of Basalt Fiber Reinforced Concrete

**DOI:** 10.3390/ma13061362

**Published:** 2020-03-17

**Authors:** Hao Zhou, Bin Jia, Hui Huang, Yanling Mou

**Affiliations:** Department of Civil Engineering and Architecture, Southwest University of Science and Technology, Mianyang 621000, China; zhouhao931124@126.com (H.Z.); huanghui871005@sina.com (H.H.); m19981750342@163.com (Y.M.)

**Keywords:** basalt fiber, concrete, mechanical properties, fiber content, toughness, crack resistance

## Abstract

Blending a certain proportion of basalt fiber into concrete improves the toughness of concrete, which prevents cracking and avoids the brittle behaviors. In this paper, the compressive, tensile, and flexural tests of concrete with different basalt fiber contents were carried out. Then the test phenomena, failure modes, and mechanical properties were compared and analyzed to derive the relationship between the basalt fiber contents and mechanical properties. The toughness and crack resistance performance of basalt fiber reinforced concrete were evaluated by the fracture energy, advanced toughness parameters, and characteristic length proposed by Hillerborg. The correlation coefficient of basalt fiber was introduced to establish the calculation formula for mechanical properties of basalt fiber reinforced concrete. The results indicated that basalt fiber significantly improved the toughness and crack resistance performance of concrete. The enhancing effect of the basalt fiber on the compressive strength of concrete is lower than that of tensile strength and flexural strength. Moreover, the improvement effect was the highest with the basalt fiber content was 0.3% and 0.4%.

## 1. Introduction

Concrete has the advantage of high compressive strength, superior corrosion resistance, and relatively lower cost, therefore, it is still the most widely used building material in the world. However, as one type of artificial brittle materials, concrete is relatively insufficient in terms of tensile strength, bending resistance, impact resistance, and toughness. As the service time increases, the defects of concrete become increasingly prominent, which leads to many problems in engineering applications.

At present, it is internationally recognized that fibers are able to be added to concrete to overcome the defects of concrete. In the past two decades, blending fibers into concrete has been widely applied in some projects [1,2,3,4,5,6], such as railway sleepers, dams, and airport sidewalks. Researchers had also investigated the fiber concrete by blending it with polypropylene fiber, polyvinyl alcohol fiber, steel fiber, glass fiber, and carbon fiber in order to improve the tensile strength, bending resistance, and impact resistance of concrete. Accordingly, the toughness and crack resistance of concrete had been improved [7,8,9,10,11].

Commonly the composite material theory and the fiber spacing theory are adopted to explain the reinforcement mechanism of fiber reinforced concrete. The former is built on the mixing rule of composite materials and the latter is the perfect bond theory between fiber and matrix. They explain the reinforcement effect of fiber on concrete from different perspectives. The theory of composite materials [12] regards the strength and elastic modulus of fiber, concrete as a superposition, to improve the performance of concrete. The theory is based on that the matrix is isotropic and homogeneous, the fibers are distributed parallel to the direction of stress, and there is no relative slip between the fibers and the matrix. However, to get the result of the enhanced function, the elastic modulus and strength of fiber must be greater than that of the matrix. Under this condition the larger the volume content of the fiber is, the more obvious the reinforcement effect will be. But the ideal result is conflicted with the existing research results [13,14,15,16,17]. The theory of fiber spacing is based on the linear elastic fracture mechanics. It is considered that there are micro-cracks and defects in the concrete. Under the action of the external force, the stress concentration is generated, which cause the crack to expand. The crack propagation can be effectively inhibited by the incorporation of fiber. It is assumed that the fiber is regularly and uniformly distributed in the chessboard-shape along the tension direction in concrete, the crack is formed in the center of the area surrounded by the fiber. Then the adhesive force between fiber and concrete is produced under the action of external force to enhance the function. The premise of the fiber spacing theory is the uniform distribution of the fibers, so that the crack resistance will be inversely proportional to the average spacing. Otherwise this theory will be partly or entirely unreliable. Therefore, the crack-resistance performance of the fiber reinforced concrete depends on the dispersion of the fiber in the concrete. Meanwhile the volume content of the fiber is also a vital factor that affects the dispersion.

Basalt fiber has significantly different characteristics from other high-tech fibers [18,19,20,21,22]. The raw material of basalt fiber originates from natural volcanic rock, which incorporates high chemical and thermal stability and produce no harmful gas or waste residue in the fiber production process. It is a kind of new green material that meets the requirements of environmental protection; the strength of basalt fiber is much higher than that of natural fiber and synthetic fiber. In addition, its elastic modulus is similar to that of carbon fiber, both higher than that of other fibers. Though their comprehensive performance is parallel, the cost of basalt fiber is lower than one-tenth of carbon fiber.

Moreover, as a multifunctional fiber, basalt fiber possesses comprehensive and excellent quality: acidic and alkaline resistance [23,24,25,26], low and high-temperature resistance, excellent wettability. Due to its three-dimensional molecules, it has higher compressive strength and shear strength compared with one-dimensional linear polymer fibers; it also has excellent suitability and aging resistance in harsh environments. The related research results show that basalt fiber is able to play an important role in enhancing the toughness and preventing cracks of concrete, mainly in the following three aspects. First, basalt fiber reinforces the concrete in a microscopic perspective and acts as a bridge at the cracks with its high elastic modulus and tensile strength [13,27]. As well, it can inhibit crack propagation, increasing the energy absorption capacity of concrete and improving the toughness of concrete [14,15,16]. Then fibers and concrete are exceptionally combined when basalt fiber excellently disperse in concrete. Thus, the reinforcement effect of fiber reinforced concrete was increased, and the pulling ability of fibers was limited [28,29]. Besides, as an inorganic material, basalt fiber has high interfacial bonding strength with concrete [30]. However, there is a certain difference in the optimal basalt fiber contents because of the physical and mechanical properties of fibers. Meanwhile, test results demonstrate some differences with the absence of related toughness evaluation index.

There are relatively many researches on the volume content of basalt fiber. In this paper, the representative research results on the optimum content of basalt fiber reinforced concrete in recent ten years are shown in the Table 1.

The research results show that the reinforced effect of basalt fiber in compressive strength is not obvious, but that in terms of tensile resistance and flexural properties resistance are better. As can be seen from the above table, most of the research on tensile properties resistance adopts split tensile test. For direct tensile test, due to the limited conditions required by the test, the related research is relatively few. At the same time, the dispersion of the optimum volume content is relatively large, the main reason is that the evaluation method of basalt fiber reinforced concrete is lack of relevant unified method, the experiment of related properties is relatively single, there are also differences in the pouring method of basalt fiber reinforced concrete and the physical parameters of the selected fiber.

In this paper, the basalt fiber content was adopted as a single variable to study the influence of basalt fiber content on the mechanical properties of concrete. Combined with test results, the toughness and crack resistance performance of basalt fiber reinforced concrete were evaluated by the fracture energy, advanced toughness parameters, and characteristic length proposed by Hillerborg and the best volume content was obtained. Meanwhile the correlation coefficient of basalt fiber was introduced to establish the calculation formula for mechanical properties of basalt fiber reinforced concrete.

## 2. Materials and Experiments

### 2.1. Specimens Preparing

According to GB/T 50081-2002 “Standard for test method of mechanical properties on ordinary concrete” and CECS 13-2009 “Test method for steel fiber reinforced concrete”. Three kinds of specimens were prepared for compression test (150 mm × 150 mm × 150 mm), tensile test (150 mm × 150 mm × 550 mm), and bending test (150 mm × 150 mm × 550 mm). The volume content of basalt fiber with respect to concrete were selected as 0%, 0.1%, 0.2%, 0.3%, 0.4%, 0.5%, 0.6%. The samples were divided into seven groups, numbered B0, B1, B2, B3, B4, B5 and B6, respectively. Three specimens were selected for each group of repeated tests, namely 63 specimens.

**Figure 1:**
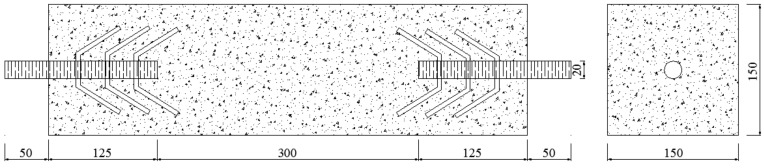
Specimen of tensile test.

For the tensile test of concrete, the two ends of the specimen were pre-embedded with rebar (20 mm in diameter, 125 mm in buried depth and 50 mm in exposed nail length) according to GB/T 50081-2002 ”Standard for test method of mechanical properties on ordinary concrete” and CECS 13-2009 “Test method for steel fiber reinforced concrete”. In order to prevent producing stress concentration near the embedded bar, the embedded part was tied with claw type vacuum wires above the front, as shown in Figure 1 (units: mm).

According to *JGJ/T 221-2010* ”Technical specification for application of fiber reinforced concrete”, we selected P∙O 42.5 Portland Cement, the sand from the river in Mianyang, China, whose modulus of fineness was about 2.5 (sand ratio 30%), local ordinary gravel stones (16 to 20 mm), and the local tap water. The water-cement ratio of cast-in-place concrete was 0.42, and the collapse degree is 30–50 mm. according to the *SL677-2014* “Specification for Hydraulic Concrete Construction”. The concrete’s mixture ratio was shown in Table 2. The specific parameters of basalt fiber were shown in Table 3.

The preparation, pouring, and curing of concrete specimens were in accordance with *GB/T 50080-2016* “Standards for Test Method of Performance on Ordinary Fresh Concrete” and *CECS 13-2009* “Standard Test Method for Steel Fiber concrete”. A forced mixer was used to mix BFRC (basalt fiber reinforced concrete). The secondary mixing method was adopted to avoid fiber agglomeration. First, fine aggregate and basalt fiber were mixed dryly for 30 s until they were evenly mixed, and then coarse aggregate, cement, and water were put in. All materials were stirred for 280 s after putting in. Vibro-compaction moulding technology was used to pour concrete into the moulds for moulding. After 24 h, the specimens were removed into the Standard Curing Room for curing.

### 2.2. Loading Test

According to *GB/T 50081-2002* “Standards for Test Method of Mechanical Properties on Ordinary Concrete” and *CECS 13-2009* “Standard Test Methods for Steel Fiber Concrete”, loading rates of compressive, tensile, and bending tests were 11.25 kN/s, 0.04 mm/min, and 0.01 mm/min, respectively.

**Figure 2:**
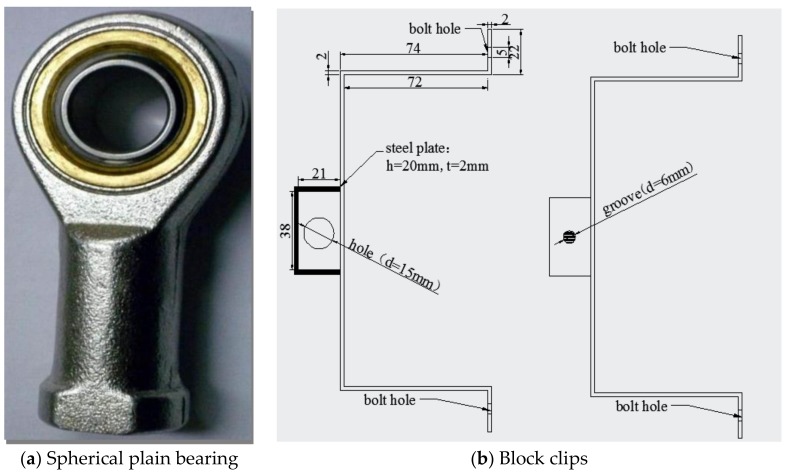
The test device.

In the tensile test, the main difficulty is the axis alignment of specimen clamping. Therefore, the pre-embedded bars at the end of the test piece were connected to the load testing machine through the Spherical Plain Bearing, as shown in Figure 2. Since the spherical plain bearing was made of high-strength steel, it can rotate freely, ensuring the axial alignment of the stressed specimens. In terms of test measurement, the Block Clips (Figure 2) were designed on both sides of the specimen for installing two LVDTs (Linear Variable Differential Transformer) to measure the displacement. Two strain gauges (100 mm×3 mm) were attached to the side of the specimen to monitor the surface cracking of the specimen and find the initial crack point of the specimen. The strain of the specimen was taken as the average of strain value on two opposite sides.

## 3. Results

### 3.1. Failure Mode

#### 3.1.1. Compressing Failure Mode

**Figure 3:**
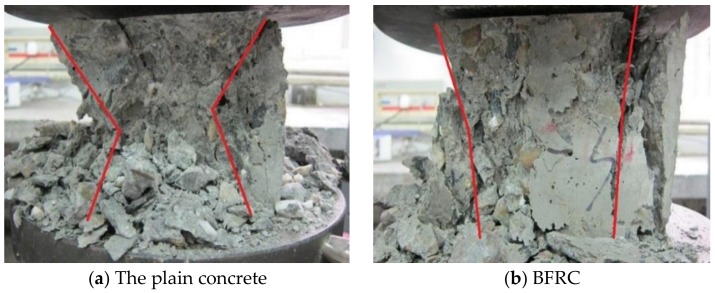
The compressing failure mode.

With the increase of load, the plain concrete specimen (B0) was crushed with a loud noise. Surrounding concrete was crushed and spalled after maximum load due to the cyclo-hoop effect, and concrete block was pyramidal, as shown in Figure 3.

For the BFRC (B1, B2, B3, B4, B5, and B6), the crack width increased gradually after cracking with debris fell. The cross sectional area showed an external drum shape. However, the specimen cracked but was not broken. Their destruction was not as sudden and quick as that of B0. The first sound of destruction was noisy and tearing and finally destruction was followed by a dull sound (Figure 3).

#### 3.1.2. Tensile Failure Mode

**Figure 4:**
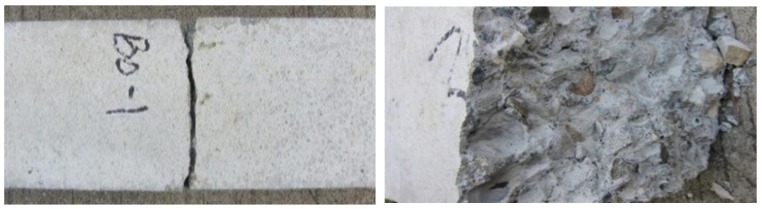
The tensile failure mode of the plain concrete.

For the plain concrete specimen (B0), the load increased steadily along with the increase of displacement in the early stage of the test. When the deformation was close to the ultimate displacement, the increase amplitude of the load value had no obvious change. When macroscopic cracks appeared in the middle of the specimen, the specimen broke into two sections, the cracking process was extremely fast, and the section was smooth and clear (Figure 4).

**Figure 5:**
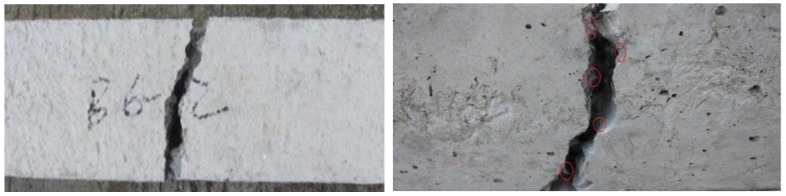
The tensile failure mode of BFRC.

For the BFRC (B1, B2, B3, B4, B5, B6), the load increased linearly with the increase of displacement at the initial stage of loading. When the deformation approached the ultimate displacement, the growth rate of the load slowed down. When macroscopic cracks were observed in the middle of the specimen, the specimen was broken into two sections with clear but uneven fracture. Similar to the case of plain concrete, the fracture sound of BFRC was smaller and there were a lot of tensional fibers in the fracture surface. Because the elastic modulus of basalt fiber was too low to bear the load effectively, the tensile load was close to zero. The specimen continued to be stretched, the fibers at the fracture were observed to be pulled off after the fracture, which indicated that basalt fiber had a fine bond performance with concrete (Figure 5).

#### 3.1.3. Bending Failure Mode

**Figure 6:**
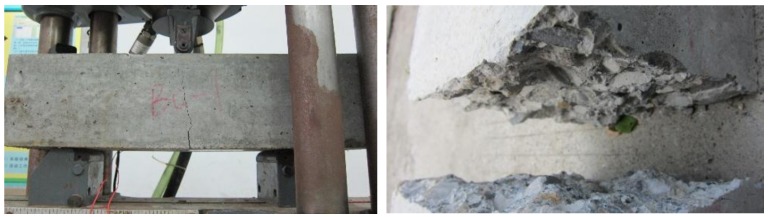
The bending failure mode of the plain concrete.

**Figure 7:**
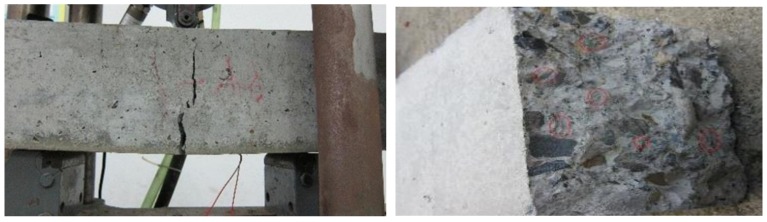
The bending failure mode of BFRC.

In the process of bending test, the failure of plain concrete (B0) was very sudden and rapid. With the increase of load, the specimen was quickly break into two parts after cracks appear. However, for BFRC there was a certain buffer time from the appearance of cracks to the fracture. Although time was very short, it proved that the toughness of concrete was indeed improved. In terms of crack width, it can be obviously observed that the crack width of BFRC was larger than that of plain concrete (Figure 6 and Figure 7).

### 3.2. Performance Parameters of Compressing and Bending

**Figure 8:**
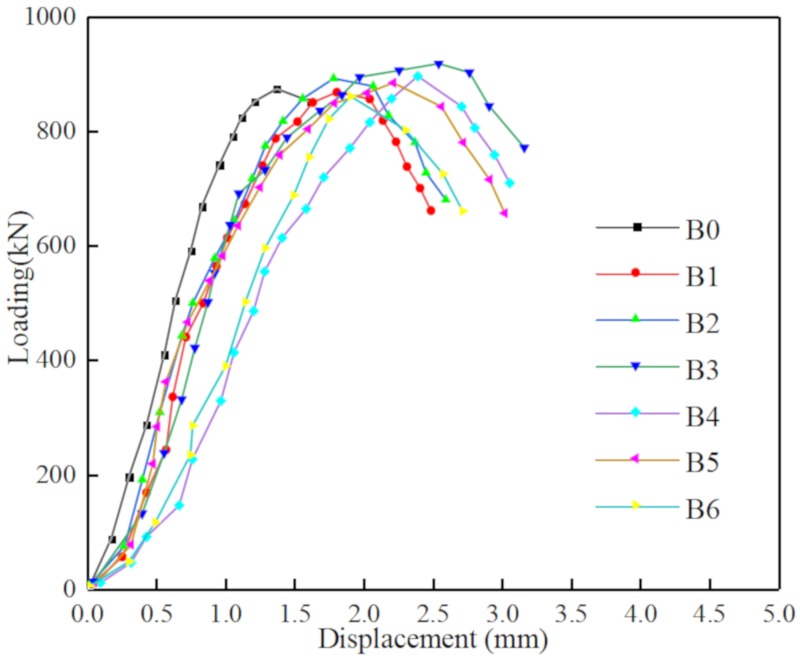
The Loading-Displacement curve of compressive test.

**Figure 9:**
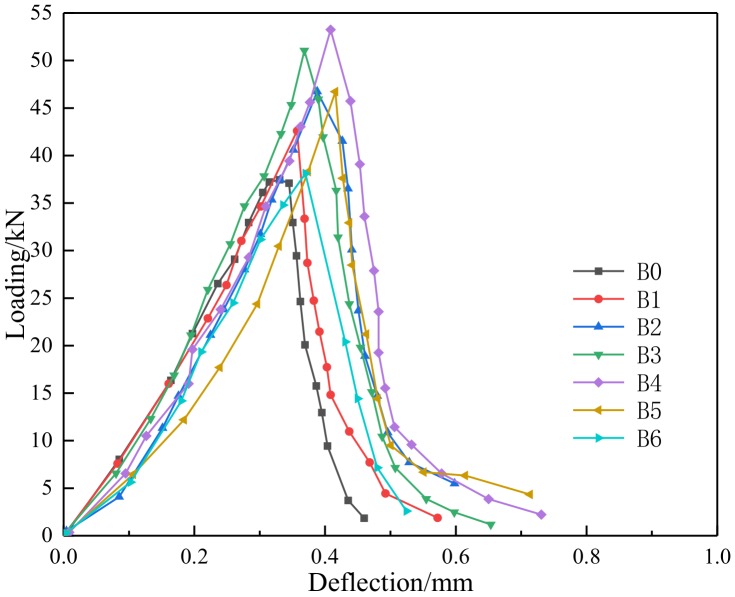
The Loading-Deflection curve of bending test.

Through compressing test and bending test, the mechanical properties parameters of BFRC can be obtained as shown in Table 4, Figure 8 and Figure 9.

#### 3.2.1. Compressive Strength and Bending Strength

**Figure 10:**
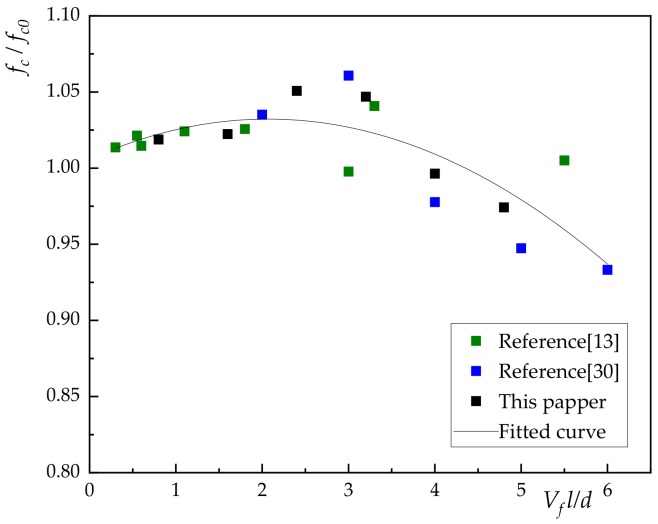
The relationship between fiber characteristic value and compressive strength ratio.

**Figure 11:**
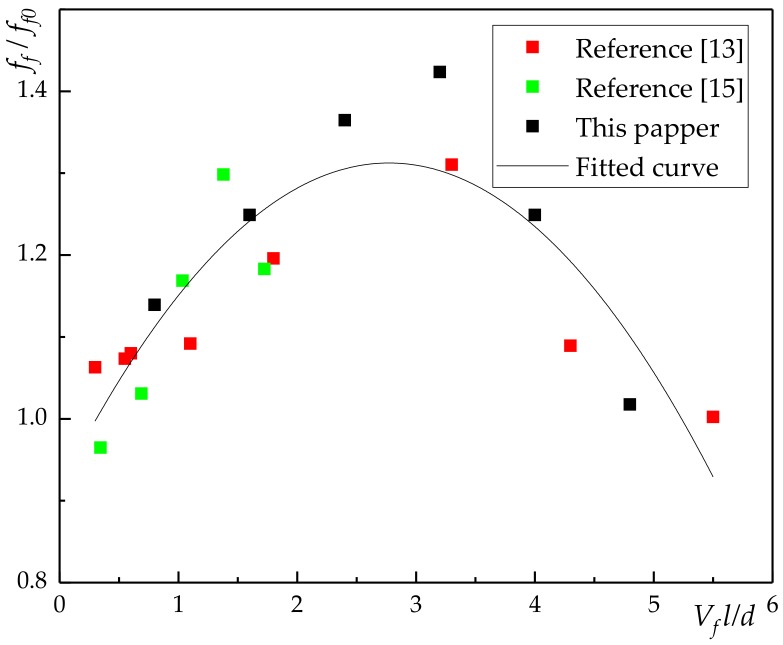
The relationship between fiber characteristic value and flexural strength ratio.

Due to the limited data in this paper, the relationship between compressive strength ratio, flexural strength ratio and fiber characteristic value ($l\frac{V_{f}}{d})$ were obtained by quoted reference [13,15,30] in Figure 10 and Figure 11.

The relationship between compressive strength and volume content of basalt fiber was given by:

(1) $$f_{c}{=f}_{c0}{[1+\lambda }_{11}l\frac{V_{f}}{d}{+\lambda }_{12}{(l\frac{V_{f}}{d})}^{2}]$$

where *f_c0_* is compressive strength of the plain concrete; *l* is the length of basalt fiber; *d* is diameter of basalt fiber; $\lambda_{1}$ is the influence coefficient of compressive strength related to fiber dispersion and pouring process, as shown in Figure 10, $\lambda_{11}$ = 0.025, $\lambda_{12}$ = −0.006.

It can be seen from Equation (1) that there was a poor agreement with fitted curve and experiment data. The results show that the compressive strength of concrete has not been significantly improved by the incorporation of basalt fiber.

Relationship between bending strength and volume content of basalt fiber:

(2) $$f_{f}{=f}_{f0}{[1+\lambda }_{21}l\frac{V_{f}}{d}{+\lambda }_{22}{(l\frac{V_{f}}{d})}^{2}]$$

where *f_f0_* is bending strength of the plain concrete; *γ_2_* is the influence coefficient of bending strength related to fiber dispersion and pouring process, as shown in Figure 11,${\;\lambda }_{21}$ = 0.286, $\lambda_{22}$ = −0.052.

Figure 10 and Figure 11 showed that the incorporation of basalt fiber can improve the bending strength and compressive strength of concrete. In addition, the effect of volume content on compressive strength was less than that of volume content flexural strength. In general, when the volume content of basalt fiber is 0.3%, the compressive strength of concrete increased by 5.07%. When the volume content of basalt fiber was 0.4%, the bending strength of concrete increased by 42.34%.

From Figure 10 and Figure 11, it can be seen that the fitting result of 0.1%–0.4% was significantly better than that of 0.5% and 0.6% under the working condition in this paper. The latter also had a certain gap with the relevant literature [13,15,30], which is mainly due to the pouring process of fiber reinforced concrete and the difference of fiber itself. Therefore, in this paper, only 0.1%–0.4% was included in the analysis of toughness and compression ratio of BFRC.

#### 3.2.2. Evaluation of Compressive Toughness

**Figure 12:**
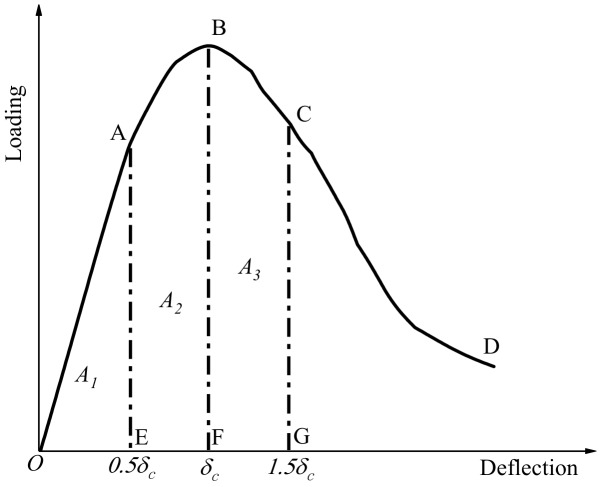
Advanced evaluation method of toughness.

The ductility of concrete is the ability to maintain deformation after cracking, that is, the area enclosed by the load-displacement curve and *x*-coordinate. Combined with the methods of *ASTM-C1080* and *JSCE-SF4*, an advanced toughness evaluation method was proposed in this paper. The peak deflection of 0.5, 1.0, and 1.5 times was selected as the reference deflection, i.e., 0.5*δ_c_*, *δ_c_*, 1.5*δ_c_*. Accordingly, the toughness was evaluated by the area of *A_1_*, *A_2_*, and *A_3_*, as shown in Figure 12; the toughness was represented by *I*_0_ and *I*_1_, see Equations (3) and (4). In addition, the specimen had been damaged and had basically lost its bearing capacity under the larger axial displacement. Therefore, there was no significance to make the further analysis and discussion here.

The results (Table 4) showed that basalt fiber can significantly improve the toughness of concrete. At first, the toughness index of concrete increased, then decreased with the increase of volume content. When the volume content was 0.4%, the toughness index *I*_0_ reached maximum; the volume content was 0.3%, the toughness index *I*_1_ was the largest.

In Figure 12, *δ_c_* was the displacement corresponding to the peak load; *A_1_* was the area which was formed by curve OA and X axis, that was area of polygon OAE; *A_2_* was the area which was formed by curve AB and X axis, that was the area of polygon ABEF; *A_3_* was the area for curve BC and X axis which was the area of polygon BCFG.

(3) $$I_{0}=\frac{A_{1}{+A}_{2}}{A_{2}}$$

(4) $$I_{1}=\frac{A_{1}{+A}_{2}{+A}_{3}}{A_{1}}$$

**Figure 13:**
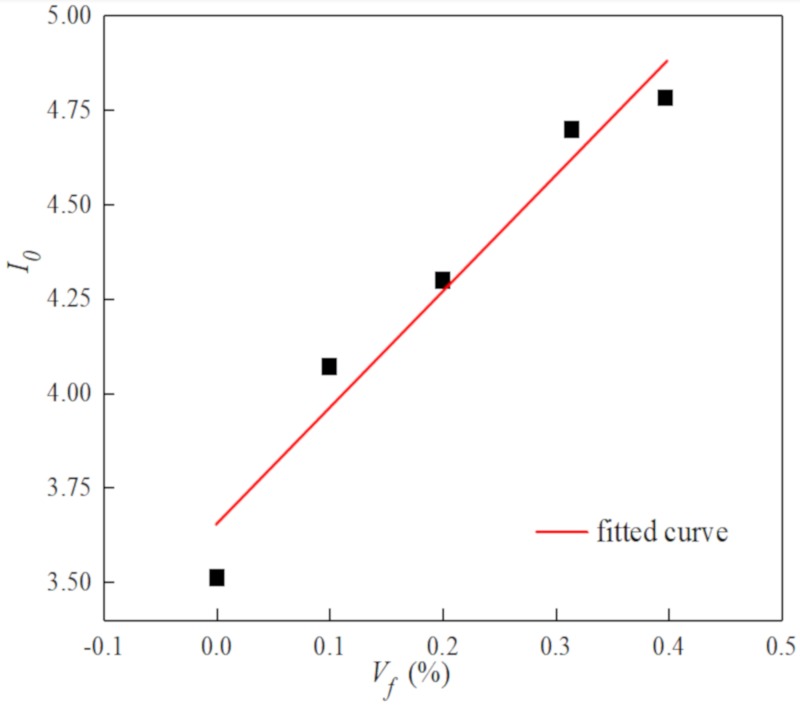
The relationship between the volume content and toughness index *I_0._*

**Figure 14:**
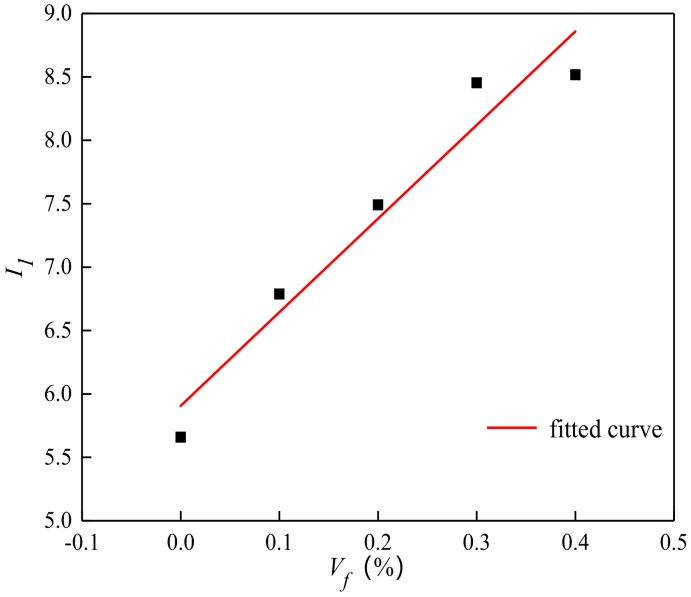
The relationship between the volume content and toughness index *I_1_***.**

The relationship between volume fraction and toughness index was shown in Figure 13 and Figure 14.

The relationship between toughness index and volume content was expressed as:

(5) $$I_{0}{=I}_{00}{(1+\beta }_{0}V_{f}\frac{l}{d})$$

(6) $$I_{1}{=I}_{10}{(1+\beta }_{1}\beta_{0}V_{f}\frac{l}{d})$$

where *I_00_* and *I_10_* are toughness index of the plain concrete. *β_0_* and *β_1_* is the influence coefficient of toughness index related to fiber dispersion and pouring process, as shown in Figure 13 and Figure 14, *β_0_* = 0.112%, *β_1_* = 1.455.

According to the Equation (5), Equation (6) and Figure 14, the toughness of BFRC increased linearly with the increase of volume content before cracking. After cracking, toughness of BFRC gradually reached the critical volume content, then the growth slowed down slightly.

#### 3.2.3. Evaluation of Bending Toughness

Bending toughness of BFRC can be evaluated by the fracture energy (*G_f_*). This paper is based on the calculation of the area of the peak load deflection under 1.2δ*_f_* curve, as shown in Table 3. The results showed that the fracture energy firstly increased and then decreased with the increase of volume content. When the volume content was 0.4%, the fracture energy was the largest.

**Figure 15:**
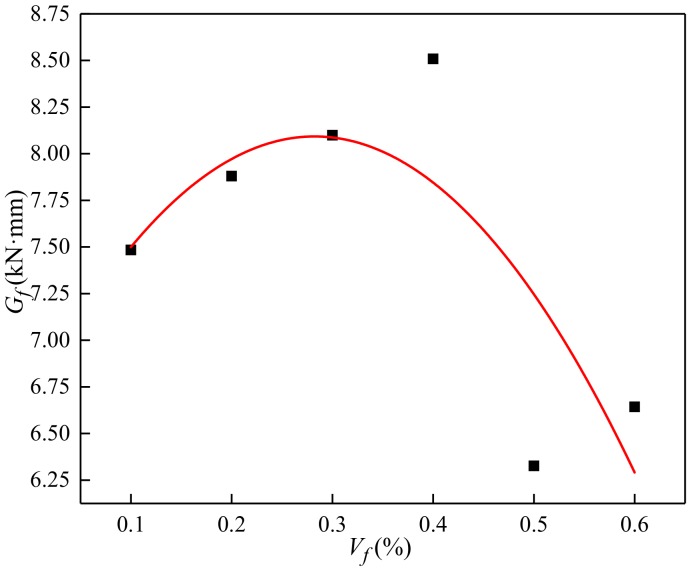
The relationship between the volume content and fracture energy.

The fracture energy was calculated by introducing the deflection influence coefficient *η_f_*. The relationship between the volume content and the fracture energy was given by Figure 15 and Equation (10).

(7) $$G_{f}=\int_{0}^{1.2\delta }{(P}_{f}+\frac{\mathrm{mg}}{1000}{)d\eta }_{1}\delta$$

(8) $$P_{f}=\frac{f_{f}{\mathrm{bh}}^{2}}{L}{=f}_{f0}{[1+\lambda }_{21}l\frac{V_{f}}{d}{+\lambda }_{22}{(l\frac{V_{f}}{d})}^{2}]\frac{{\mathrm{bh}}^{2}}{L}$$

(9) $$G_{f}=\int_{0}^{1.2\delta }\left[f_{f0}{[1+\lambda }_{21}l\frac{V_{f}}{d}{+\lambda }_{22}{(l\frac{V_{f}}{d})}^{2}]\frac{{\mathrm{bh}}^{2}}{L}+\frac{\mathrm{mg}}{1000}\right]{d\eta }_{1}\delta$$

(10) $$G_{f}=\frac{{\mathrm{bh}}^{2}}{L}\int_{0}^{1.2\delta }\eta_{1}f_{f0}d\delta +\int_{0}^{1.2\delta }\eta_{1}\frac{\mathrm{mg}}{1000}d\delta +\frac{{\mathrm{bh}}^{2}}{L}\int_{0}^{1.2\delta }\eta_{1}f_{f0}{[\lambda }_{21}l\frac{V_{f}}{d}{+\lambda }_{22}{(l\frac{V_{f}}{d})}^{2}]d\delta .$$

where *L* is distance between supports at the bottom of the bending test piece; mg is the gravity of specimen; and $\eta_{1}$ = 0.4571, it is related to fiber dispersion and pouring process.

The fracture energy fully reflected the energy consumption in the process of fracture. The higher the fracture energy was, the more obvious the effect was. The incorporation of basalt fiber had the most obvious effect on the crack resistance of concrete. The effect of fiber incorporation on deflection is greater than that on strength.

#### 3.2.4. Bend-Press Ratio

There was a strong correlation between the compressive strength and bending strength of concrete, and the bend-press ratio is a constant value for the same grade of concrete. However, the bend-press ratio must be different due to the different volume of basalt fiber. Therefore, this paper firstly calculated the bend-press ratio in plain concrete by Equation (8). Based on the bend-press ratio of plain concrete, the enhancement coefficient was introduced and finally the equation for bend-press ratio of BFRC was obtained by Equation (9).

(11) $$\frac{f_{f0}}{f_{c0}}=0.13$$

(12) $$\frac{f_{f}}{f_{c}}=0.13\alpha$$

**Figure 16:**
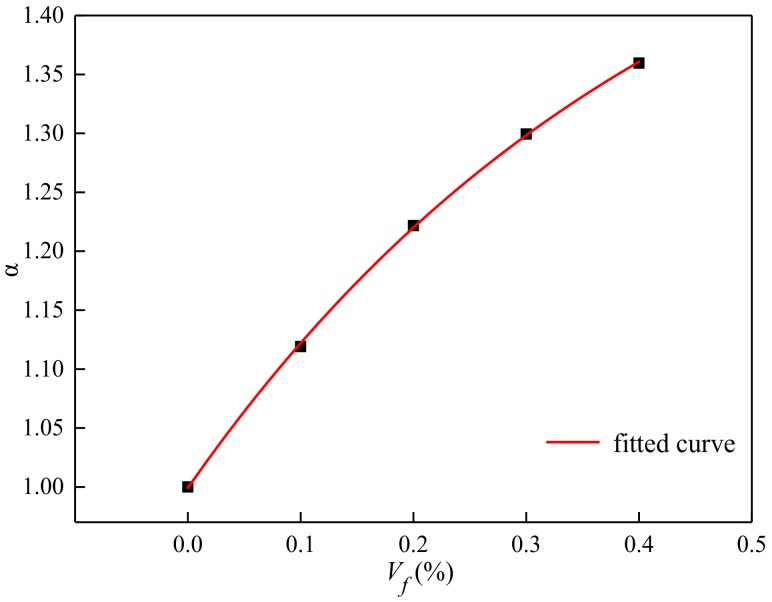
The relationship between the volume content and enhancement coefficient.

The relationship of enhancement coefficient and volume content was expressed by Figure 16 and Equation (10).

(13) $$\alpha =1.608\;-\;0.608\exp(-2.256V_{f})$$

Test results showed that basalt fiber can improve the crack resistance of concrete. With the increase in volume content from 0.1% to 0.4%, the bend-press ratio of BFRC was increased, obviously.

### 3.3. Tensile Performance Parameters

**Figure 17:**
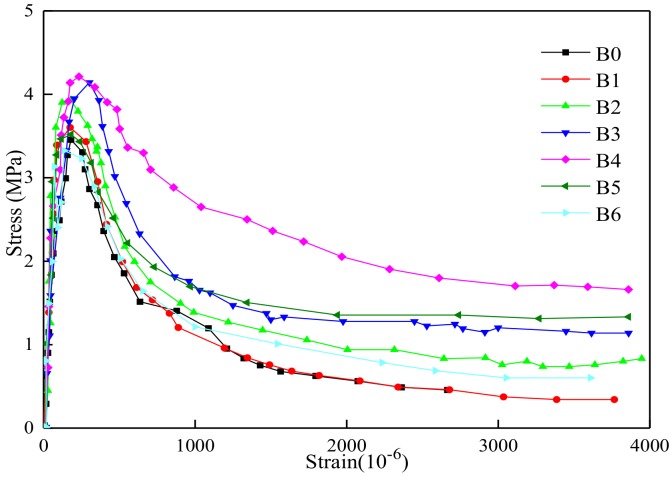
The Stress-Strain curve of tensile test.

Through the tensile test of BFRC, the full tensile stress-strain curve of BFRC was showed in Figure 17, and the tensile performance parameters were given in Table 5.

As seen in Table 5, the volume content of basalt fiber had a great influence on the tensile strength of concrete. With the increase of volume content, the tensile strength firstly increased and then decreased. When the volume content was 0.4%, the tensile strength was increased most (4.21%), the tensile elastic modulus, ultimate tensile strain, and maximum crack width were increased by 4.21%, 4.37%, 23.51%, and 37.61%, respectively. However, when the volume content was 0.5% and 0.6%, the tensile strength decreased instantaneously. The specific reason was that the dispersion of basalt fiber was too worse to result in the weak area inside concrete. In conclusion, BFRC had good toughness and crack resistance performance when the volume content was 0.4%.

#### 3.3.1. Tensile Strength

**Figure 18:**
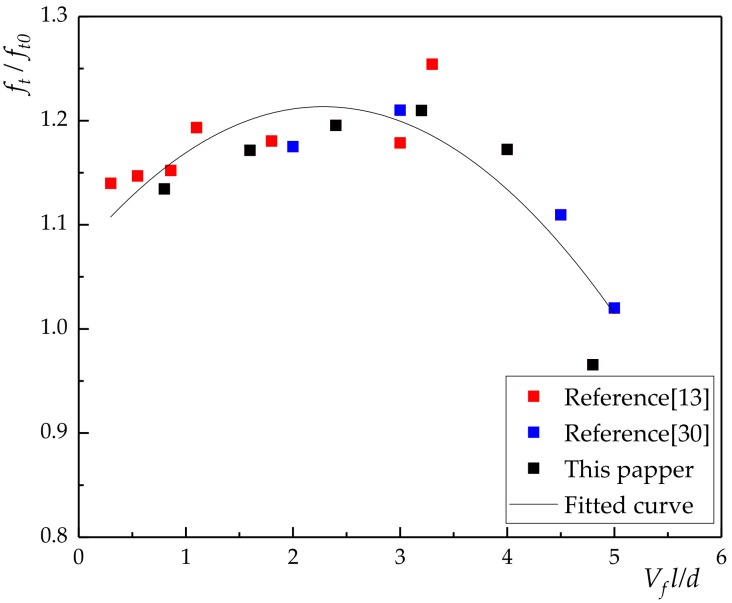
The relationship between fiber characteristic value and tensile strength ratio.

The relationship between fiber characteristic value and tensile strength ratio were drawn based on the test results (Figure 18, Equation (14)).

(14) $$f_{t}{=f}_{t0}{[1+\lambda }_{31}l\frac{V_{f}}{d}{+\lambda }_{32}{(l\frac{V_{f}}{d})}^{2}]$$

where *f_t0_* is tensile strength of the plain concrete.${\;\lambda }_{3}$ is the influence coefficient of tensile strength, as shown in Figure 18,${\;\lambda }_{31}$ = 0.123,$\lambda_{32}$ = −0.027.

For the result of relationship between elasticity modulus and volume content, it is found that the correlation coefficient was not satisfactory. Therefore, the regression equation was not listed and the specific relationship needs to be further studied.

#### 3.3.2. Crack Width

In the tensile process of BFRC, the total deformation (*δ*):

(15) $${\delta =\delta }_{e}{+\delta }_{0}+\omega$$

(16) $$\delta_{e}=\frac{\sigma_{p}}{E_{t}}l$$

(17) $$\delta_{0}{=\delta }_{p}\;{-\;\delta }_{e}$$

where *ω* is the crack expansion width, (mm); *δ_e_* and *δ_0_* are the elastic deformation and residual deformation, (mm); *σ_p_* is the peak stress, (MPa); 𝑙 is the gauge length of the specimen, (mm); *δ_p_* is the peak stress corresponding deformation (mm).

The crack width and gauge length of the specimen were irrelevant, meanwhile there were no macroscopic cracks before peak stress. From Equation (15), the crack expansion width can be expressed:

(18) $${\omega =\delta \;-\;\delta }_{e}\;{-\;\delta }_{0}$$

**Figure 19:**
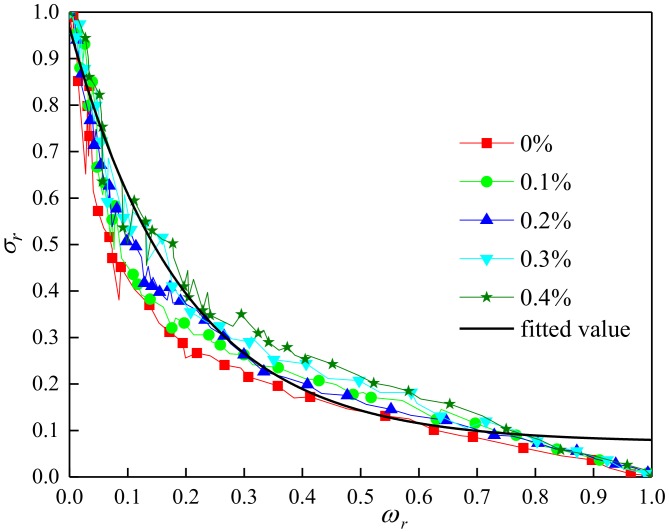
Relationship between relative tensile stress and relative crack width.

Then, the relative stress-crack width curve (Figure 19) and the new fitting Equation (22) were obtained.

The empirical equation derived by scholars [37] was as follows:

(19) $${\sigma =f}_{t}\left[1\;-\;\varphi \exp(-(|\gamma \right|\frac{\omega_{t}}{\omega }{)}^{n}]$$

And

(20) $$\sigma_{r}=\frac{\sigma }{f_{t}}$$

(21) $$\omega_{r}=\frac{\omega }{\omega_{t}}$$

With the method of fitting regression, the new expression was obtained:

(22) $$\sigma_{r}=a\;-\left(a\;-\;b\right)\exp\left[-(\left|\gamma \right|\omega_{r}{)}^{n}\right]$$

where *σ_r_* is the relative tensile strain; *ω_r_* is the relative crack width; *a* = 0.0418, *b* = 1.3082, $\left|\gamma \right|$ = 11.7864, *n* = 0.4932, *α*, *β*, $\left|\gamma \right|,\;$and *n* were all related to the type, aspect ratio, density, other physical and mechanical parameters of basalt fiber.

#### 3.3.3. Maximum Crack Width

**Figure 20:**
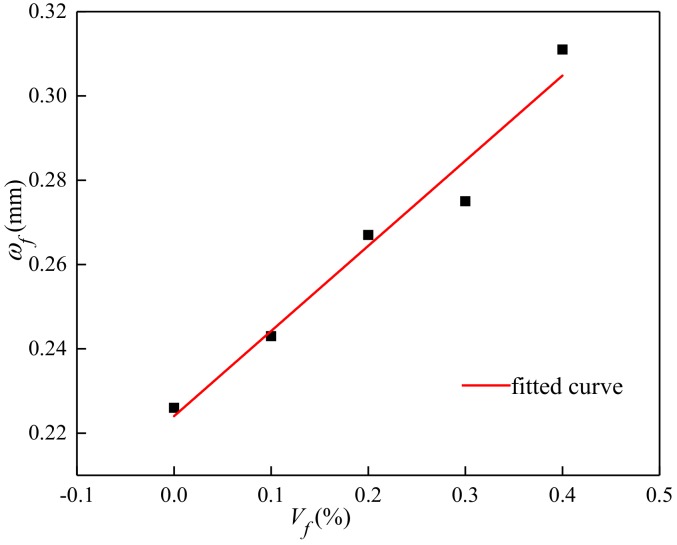
Relationship between the volume content and maximum crack width.

According to the test results in Table 4, the relationship between the volume content and the maximum crack width was given by Figure 20 and Equation (23).

(23) $$\omega_{t}{=\omega }_{0}{(1+\psi V}_{f}\frac{l}{d})$$

where *ω_0_* is maximum crack width of plain concrete; *ψ* is the influence coefficient of maximum crack width, *ψ* = 0.1173%.

#### 3.3.4. Fracture Energy

The fracture energy of concrete is an important parameter to characterize the energy consumption in the process of concrete fracture and crack propagation. The greater the fracture energy was, the more the energy consumption in the process of fracture was. The fracture energy of BFRC was more than that of plain concrete. The main reason was that the basalt fiber needed to consume energy when it was broken. This also indicated that the fracture resistance of basalt fiber was effective. Different from the above calculation method of fracture energy, another method [38] was adopted.

Fracture energy specifically referred to the energy consumption of cracks per unit area, which was shown on the curve as the ratio of the area under the load-crack width curve to the cross-sectional area of the specimen, that was, the area under the stress-crack width curve:

(24) $$G_{t}=\int_{0}^{\omega_{f}}f_{t}d\omega$$

**Figure 21:**
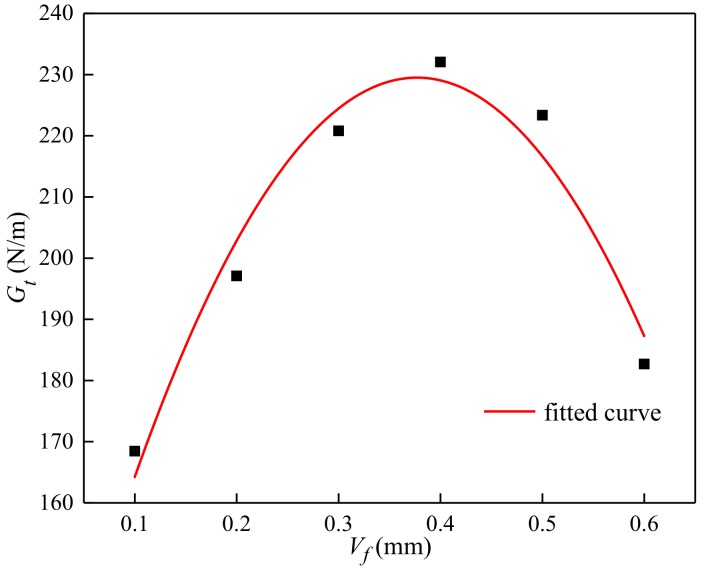
Relationship between the volume content and fracture energy.

The test results of fracture energy were shown in Table 4 and Figure 21.

It can be seen that the fracture energy of concrete increased with the increase of volume content (Figure 21). The fitted equation between specific fiber volume content and fracture energy was as follows:

(25) $$G_{t}=\mu {\;[1+\lambda }_{31}l\frac{V_{f}}{d}{+\lambda }_{32}{(l\frac{V_{f}}{d})}^{2}]\int_{0}^{\omega_{f}}f_{t0}d\omega$$

where μ is the influence coefficient of fracture energy, μ = 0.6691.

#### 3.3.5. Characteristic Length

There are many methods for characterizing the fracture properties of concrete [38]. This paper used the characteristic length proposed by Hillerborg to analyze the fracture properties of BFRC:

(26) $$L_{\mathrm{ch}}=\frac{E_{t}G_{t}}{f_{t}^{2}}$$

The specific dates of the characteristic length of each specimen were shown in Table 4. The characteristic length of concrete was negatively correlated with the brittleness of concrete. According to the test results, it can be found that when the volume content of basalt fiber increased from 0.1% to 0.4%, the characteristic length of fiber concrete increased from 15.11% to 22.63%. In other words, with the increase of basalt fiber volume content (no more than 0.4%), the characteristic length of concrete also increased. The above analysis shows that the reasonable incorporation of basalt fiber can reduce the brittleness of concrete and increase its toughness.

## 4. Discussion

As above, the related work was carried out on the mechanical properties such as strength, toughness, crack resistance, and energy dissipation under various stress states. The effect laws of volume content of basalt fiber on each mechanical property parameter were found simultaneously. Interfacial bonding reliability between concrete and basalt fiber played a tremendously essential role in potentiation. With the failure mode of fracture surface, the bonding property was authentic between basalt fiber and concrete. It can guarantee that the force applied to matrix was transferred to fiber via the surface of basalt fiber and concrete. Micro-crack of concrete developed toward macro-crack, then basalt fiber which across crack became the main undertaker of external force when the external load increased to a certain extent. Furthermore, due to higher performance for extension strength and toughness of basalt fiber more than concrete, the stress concentration at the crack tip was mitigated and the extension resistance of crack was increased. So the tension resistance and bending resistance were reinforced. Additionally, a certain amount of energy was consumed during the process of resisting the extension of crack, thereby the fragility crack was relieved at some extent.

(1) Micro-fracture of Concrete

In the early after concrete pouring, there is a possibility of concrete bleeding that many capillary bleeding channels will be formed inside naturally, some moisture will transfer to surface and then be evaporated, more, when the transfer speed is slower than evaporation, this moisture will develop from surface to the inside and curvature is going to be increased in this condition. According to capillary principle, we know that concave surface is going to be shrank under the force of inside tension, capillary wall is under tensile condition resulting from inside negative pressure, additionally, if this compression keeps increasing, then there will be crack in the concrete surface, later, many cracks will form fracture which can be the micro reason for forming micro-fracture.

Propagation of crack does matter intensity, deformation, and destructive property. Cracks which are formed before undertaking load can be divided into randomly distributed micro-crack and directional macro-crack. In the cracking, concrete is mainly under control by micro-cracks, on the one side, micro-crack will develop into macro-cracks, on the other side, it will have dual effects on main cracks on shielding and degradation.

(2) Crack Resistance Effect in the Process of Shrinkage of Concrete

There will form a three-dimensional chaotic network system which equals secondary reinforcement effect that decreases the separation between aggregate in the surface and bleeding restraining generation and development of segregation crack and stopping from developing fracture after certain basalt fiber were added into concrete. It’s easily to form shrinkage crack in the early pouring period resulting from hydration reaction and evaporation between concrete and water and lower strength of extension. Compared with plastic slurry, basalt fiber is a little bit higher in elasticity modulus, the adsorption bonding force will disperse some of the shrinkage energy to the fiber mono-filament, increasing strength of extension so that it can efficiently restrain the production and development of micro-cracks, especially for interconnected fracture, at some extent, it increases the degree of density of concrete. In addition, the fiber in the surface material increases the difficulty of water transfer, thus reducing the tension formed by capillary water loss and contraction, as well as the evaporation of water, which can improve the water retention of concrete.

(3) Crack Resistance Effect in the Process of Hardening

There are three types of shrinkage for concrete in the process of hardening: drying shrinkage, temperature shrinkage, and carbonization shrinkage. Drying shrinkage which refers to the shrinkage due to the capillary stress for evaporation of inside moisture. Temperature shrinkage can be a “thermal contraction” which forms in the process of using or the ambient temperature drops. Carbonization shrinkage refers that after hydration, due to chemical reason, the concrete will form calcium hydroxide, then the carbon dioxide will infiltrate through capillary channel and form calcium carbonate reacting with calcium hydroxide. And, the size for it is small, then can lead to shrinkage. The shrinkage above will form tensile stress and form fracture under constraint condition. The steel fiber in concrete can not only withstand the tensile stress reducing the generation of cracks, but also reduce the stress concentration at the tip of micro-cracks preventing the expansion of micro-cracks, preventing the appearance of connected cracks. When cracks are being developed, the fiber will cross crack like a bridge for interconnection between fiber and cracks and short distance, due to this transfer, which ease the stress concentration in the front of micro-crack leading to more continuous and uniform field of stress inside of concrete then restraining forward development of micro-cracks at some extent; Also, when the space of fibers is longer than micro-cracks, then fibers will form blocking effect and change its extension ways or micro-cracks will cross fibers so that they can be separated into finer crack space which does stops from producing and developing micro-cracks.

(4) Toughness Strengthening Mechanism of Basalt Fiber Concrete

According to the observation to the failure state of basalt fiber, it can be obtained that basalt fiber is mainly by means of stopping the production and development of crack to slow down its steady expansion before failure, more, it has an effect of stopping from cracks and increasing toughness through micro-crack parts or fracture transition parts in the crack tip.

Seen Figure 8, Figure 9 and Figure 17, with the content of fibers were increased from 0 to 0.4, the strength also was going to be improved, and the curve was going to be more full with higher toughness. While the load was small, the load was transferred to fiber through the bonding force between basal part and fiber surface, then basalt fiber and concrete could be one piece to undertake the load together, in this time, the deformation coordination was in the elastic stage so the load deflection curve showed straight. While, if the load kept increasing and transformation reached the initial crack strain of basalt fiber concrete, cracks were produced, so the basalt fiber which cross cracks transferred stress and made a difference from plain concrete which not stop development of cracks to keep it balanced.

With the increase of load, micro-cracks inside of basal part developed steadily into macro-cracks, then the load deflection curve was going to be nonlinear variation. In this stage, due to boding force, basalt fiber transferred stress across the fracture, and the basalt fiber concrete still could load more. More the load, more the development of cracks, then basalt fiber reinforced concrete was in the elastic-plastic stage. After the process for pulling out fiber little by little until it failure, the load capacity for it still was improved and increased more with the increase of volume content.

After basalt fiber concrete reached its critical value for load, then the cracks were developed. Resulting from the critical value of bonding force between basalt fiber and basal part, the basalt fiber was pulled out until broken leaded to failure of load capacity. The basalt fiber concrete at both ends of the crack almost turned into a rigid body and the mid-span deflection increased rapidly. In this process, as more and more volume content of basalt fibers was falling off, pulling out or broken, they needed to absorb a lot of energy, so the load deflection curve dropped slowly, showing good toughness, and had the characteristics of cracking and continuous. It can also be seen from the curve that the larger the fiber volume content was, the fuller the curve descending section was, and the higher the toughness of fiber concrete was.

While under the compressed condition, fibers along the direction of force suffered the compressed force mainly. The fiber did not offer resistance, basically, as it was with vulnerable rigidity. Other fibers in different direction had the ability to prevent crack from transverse extension, they can also improve compressing strength and toughness of concrete, consume a certain amount of energy. Nevertheless, the reinforced range was smaller than the tensile state and flexural state.

The reason why basalt fiber can perfect mechanical property of concrete was benefited from crack resistance mechanism of fiber. On the premise of excellent fiber dispersion, basalt fiber contributed to the volatilization for extra water of concrete, the formation of cavities in concrete was prevented or reduced. Meanwhile, basalt fiber with fine dispersion conquer stress concentration phenomenon due to compress, air shrinkage, and other effects, then it can stop micro crack from more extension or formation which contribute to relieving the space to form, extend crack to damage situation improving the mechanical property of concrete.

However, what is worth mentioning that each mechanical property parameters show a trend of increasing first and then decreasing with the increase of basalt fiber volume content no matter the strength, toughness, or dissipation. According to the existing theoretical results, while there was relatively low content volume of fiber, so, the force to extent speed of crack for basalt fiber would be restricted; On the contrary, while there are relatively high content volume and terrible dispersion in the concrete for basalt fiber which can be easily caused lump and then formed certain holes, at the same time, the bonding performance between fiber and concrete would be greatly weakened leading to a poor effect on improvement.

## 5. Conclusions

Compared with the compression, tensile, bending test of BFRC and plain concrete, the following conclusions were obtained:

(1) The optimum volume fraction of basalt fibers is 0.3% and 0.4% within the scope of this study. In this case, the compressive strength, tensile strength, flexural strength, toughness index, fracture energy, flexural-compressive ratio, and reinforcement coefficient of concrete are significantly improved. With the volume fraction of basalt fiber exceeding the optimum volume fraction, the mechanical properties of basalt fiber are weakened.
(2) By comparing with the tensile strength and bending strength, there was no significant improvement in compressive strength. In other words, the incorporation of basalt fiber can improve tensile strength and bending strength more than compressive strength.
(3) The failure mode of concrete can be changed by the incorporation of basalt fiber from brittle failure to non-brittle failure. In addition, by observing the failure characteristics of fibers at the failure section, it can be judged that there is a good bonding behavior between basalt fiber and concrete.
(4) The compressive toughness, the tensile toughness, and bending toughness of BFRC were evaluated by the advanced evaluation method of toughness, Hillerborg characteristic length, and fracture energy, respectively. It indicated that the incorporation of basalt fiber was an adoptable way to improve the toughness of concrete performance and crack resistance.

## Figures and Tables

**Table 1:** Research status of the optimum mix amount (*V_best_*) of basalt fiber.

Scholar	Test	Diameter	Length	*V_best_*
Wang,J. [30]	Compressive, flexural, splitting tensile	15 μm	30 mm	0.1%
Chen,F. [31]	Compressive, splitting tensile	17 μm	24 mm	0.04%
Kabay N. [32]	Compressive, flexural	13–20 μm	24 mm	0.16%
Jiang,C. [13]	Compressive, flexural, splitting tensile	20 μm	12 mm	0.3%
Kizilkanat A B. [16]	Compressive, Splitting tensile	13–20 um	12 mm	0.25%
Pehlivanh,Z.O. [33]	Compressive, flexural	/	8 mm	0.304%
Jalasutram S. [27]	Compressive, flexural, splitting tensile	13 μm	12.7 mm	0.1%
Arslan, M. E. [34]	Compressive, splitting tensile, flexural	13–20 μm	24 mm	0.07%
Branston, J. [35]	Compressive, flexural	16 μm	36 mm	0.46%
Katkhuda, H. [36]	compressive, splitting tensile, flexural	16 μm	18 mm	0.3%

**Table 2:** Concrete mix design for 1 m^3.^

Materials	Cement	Stone	Sand	Water
Weight (kg/m^3^)	330	1389.6	540.5	139

**Table 3:** Related parameters of basalt fiber.

Type	Diameter (µm)	Length (mm)	Density (g/cm^3^)	Tensile Strength (MPa)	Elasticity Modulus (GPa)	Extension at Break (%)
short-cut	15	12	2.65	3500–4500	95–115	2.4–3.0

**Table 4:** Performance parameters of specimens.

Specimen	*V_f_*	*f_c_*	*cov*	*δ_c_*	*I_0_*	*I_1_*	*f_f_*	*cov*	*δ_f_*	*G_f_*	*f_f_*/*f_c_*	*α*
B0	0	39.24	0.039	1.38	3.51	5.66	5.1	0.037	0.33	7.645	0.130	1
B1	0.1	39.98	0.024	1.81	4.07	7.35	5.81	0.047	0.35	7.455	0.145	1.12
B2	0.2	40.12	0.033	1.79	4.3	7.49	6.37	0.077	0.37	7.88	0.159	1.22
B3	0.3	41.23	0.034	2.55	4.7	8.64	6.96	0.101	0.40	8.099	0.169	1.30
B4	0.4	41.08	0.027	2.4	4.74	8.52	7.26	0.092	0.41	8.508	0.177	1.36
B5	0.5	39.10	0.030	2.22	3.95	7.15	6.37	0.142	0.42	6.326	0.163	1.25
B6	0.6	38.23	0.058	1.91	3.79	7.13	5.19	0.168	0.41	6.643	0.136	1.04

Where *V_f_* is the volume content of basalt fiber, %; *f_c_* is compressive strength, MPa; *δ_c_* is the peak displacement, mm; *I_0_* and are *I_1_* toughness index; *f_f_* is bending strength, MPa; *δ_f_* is the peak deflection, mm; *G_f_* is the fracture energy of bending test, kN·mm; *α* is enhancement coefficient of BFRC depended on fiber type, aspect ratio and volume contentment; *cov* is the coefficient of variation.

**Table 5:** Tensile performance parameters.

Specimen	*V_f_*	*f_t_*	*cov*	*E_t_*	*ε_t_*	*ω_t_*	*G_t_*	*R_G_*	*L_ch_*	*R_L_*
B0	0	3.48	0.078	18.3	181.02	0.226	162.3	0	245.25	0
B1	0.1	3.6	0.091	18.1	185.43	0.243	168.45	3.79	282.31	15.11
B2	0.2	3.88	0.048	18.7	198.75	0.267	197.1	21.44	293.8	19.79
B3	0.3	4.16	0.077	19.4	212.32	0.275	220.8	36.04	297.03	21.11
B4	0.4	4.21	0.067	19.1	223.57	0.311	232.05	42.98	300.08	22.36
B5	0.5	3.54	0.118	19.3	201.02	0.249	223.35	37.62	257.99	5.19
B6	0.6	3.36	0.091	17.9	184.50	0.229	182.7	12.57	231.74	-5.51

Where *f_t_* is the tensile strength, (MPa); *E_t_* is the tensile elastic modulus, (GPa); *ε_t_* is the ultimate tensile strain, (με); *ω_t_* is the maximum crack width; *G_t_* is the fracture energy, (N/mm); *R_G_* is the increasing rate of fracture energy, (%); *L_ch_* is the characteristic length, (mm); *R_L_* is the increasing rate of characteristic length, (%).
